# Ignorance may be Bliss (for Intensivists), but not for ICU Patients!

**DOI:** 10.5005/jp-journals-10071-23143

**Published:** 2019-04

**Authors:** Sumitra G Bakshi, Atul P Kulkarni

**Affiliations:** 1 Department of Anaesthesiology, Critical Care and Pain, Tata Memorial Hospital, Homi Bhabha National Institute, Mumbai, Maharashtra, India; 2 Division of Critical Care Medicine, Department of Anaesthesiology, Critical Care and Pain, Tata Memorial Hospital, Homi Bhabha National Institute, Mumbai, Maharashtra, India

## Abstract

**How to cite this article:** Bakshi SG, Kulkarni AP. Ignorance may be Bliss (for Intensivists), but not for ICU Patients! Indian J Crit Care Med 2019;23(4):161–162.

Pain is the most ignored entity, by the intensivists, in the critically ill patients. Untreated pain in critically ill patients makes them vulnerable to chronic pain and stress disorder and may lead to poor outcomes.^1,2^ Once considered indicators of pain, hemodynamic parameters are proven to be inaccurate reflectors of pain.^2^ Identifying and treating pain in the intensive care unit (ICU) patients who have altered mentation, are sedated, and on ventilators, is challenging.^3^ As pain is subjective, patient's description and characterization of pain is essential for effective treatment.^3^ Identifying presence of pain and incorporating objective pain assessment tools in the ICU is essential. Amongst the available tools for assessment of pain, the most validated tools remain the behavioral pain scale (BPS) and critical-care pain observation tool (CPOT).^3^ Khoddam and colleagues, in this issue of the journal, describe the impact of instituting CPOT on pain assessment and management in their ICU.^4^

Critical-care pain observation tool is an assessment tool for use in the ICU in patients who are unable to communicate.^5^ It includes four sections; facial expression, body movements, compliance with the ventilator for intubated patients, and muscle tension. Previous studies have shown good psychometric properties of the tool and its role in assessing the effectiveness of analgesic treatment in the critically ill.^5^ BPS is another validated tool available for pain assessment in the ICU. It has three subscales: facial expression, upper limb movement, and compliance with mechanical ventilation.^2^ The BPS and the CPOT have common parameters which include facial expression, body movement, and ventilator compliance.^6^ Preliminary studies looking at a combination of both scales suggest that summing of the two scores may reflect better pain assessment than either of the individual scales. However, further studies are warranted to examine this combination.^6^

Nurses play a critical role in pain management in the ICU. It has been seen that though the nurses follow a “protocol-based” nursing practice for ventilated patients, a pain management plan is missing. Since, the nurses have adequate opportunity to assess, identify, and evaluate pain; they would be expected to play an active role in pain management.^7^ However, studies have shown that ICU nurses report less pain and around 60% of patients in certain centers do not receive analgesics during painful procedures.^8^ Validated tools including BPS or CPOT are not commonly used in ICUs across the globe.^9^

While exploring the experience of ICU nurses with the use of pain assessment scales, Deldar and colleagues found organizational and attitudinal barriers along with knowledge-related barriers.^7^ Physician inattention to pain management decreased the nurses attention to pain assessment and relief.^7^ Suboptimal nurse to patient ratio and heavy workload on the nurses tends to draw their attention from less emphasized duties.^7^ Lack of knowledge also contributed to the problem. Pain was not considered a serious issue and some of the nurses had limited faith in pain scales.^7^

There are lessons that can be learnt from quality improvement programs specifically focused on use of pain scales in the ICU.^9^ An outstanding example is from the Raigmore Hospital initiative.^9^ An analgesia first approach—model for improvement utilizing the CPOT was enforced. Over a 6-month period, the project using quality improvement tools and techniques emphasized on implementation of the CPOT. Result of this project was welcomed and it showed an 88% increase in 4 hourly assessments of pain. Analgesic administration based on CPOT scores increased to 100%.^8^ Khoddam and colleagues report similar findings.^4^ The study was carried out in three phases. Initially patient data such as physiological parameters, number of pain assessments by the nurses, and details of analgesics prescribed was extracted from ICU records. In the interventional phase 60 nurses were included. All nurses were trained in a 1-day educational workshop about patients’ pain documentation and use of the COPT. In the post implementation phase patients’ medical files were rescrutinized for data similar to preintervention phase. A significant increase in nurses’ pain assessment per patient was seen with a statistically significant increase in number of analgesics prescribed after intervention. However, impact of the improvement in this assessment was not assessed in this study. Taking cue from available literature, we hope that we get further studies focusing on the impact of the change in nursing practice on clinically useful endpoints such as duration of mechanical ventilation, length of ICU stay, mortality, and complications.^1^

Pain management in ICU continues to be a healthcare concern.^4^ The best possible management of pain is feasible if nurses take up this challenge and embrace the accountability for pain management.^10^ Nurses traditionally are patients’ advocates and it is time for the nurse educators to impart the necessary knowledge to their future generations to play their legitimate role. Nurses can then become competent, confident, and equipped to manage pain effectively in all clinical areas.^10^
